# Cryptic diversity and limited connectivity in octopuses: Recommendations for fisheries management

**DOI:** 10.1371/journal.pone.0214748

**Published:** 2019-05-13

**Authors:** Annelore Hilde M. Van Nieuwenhove, Hajaniaina Andrianavalonarivo Ratsimbazafy, Marc Kochzius

**Affiliations:** 1 Marine Biology, Ecology and Biodiversity, Vrije Universiteit Brussel (VUB), Brussels, Belgium; 2 Institut Halieutiques et des Sciences Marines, Université de Toliara, Toliara, Madagascar; University of California Santa Cruz, UNITED STATES

## Abstract

The market demand for octopus grows each year, but landings are decreasing, and prices are rising. The present study investigated (1) diversity of Octopodidae in the Western Indian Ocean (WIO) and (2) connectivity and genetic structure of *Octopus cyanea* and *O*. *vulgaris* populations in order to obtain baseline data for management plans. A fragment of the cytochrome C oxidase subunit 1 (COI) gene was sequenced in 275 octopus individuals from Madagascar, Kenya and Tanzania. In addition, 41 sequences of *O*. *vulgaris* from South Africa, Brazil, Amsterdam Island, Tristan da Cunha, Senegal and Galicia were retrieved from databases and included in this study. Five different species were identified using DNA barcoding, with first records for *O*. *oliveri* and *Callistoctopus luteus* in the WIO. For *O*. *cyanea* (n = 229, 563 bp), 22 haplotypes were found, forming one haplogroup. AMOVA revealed shallow but significant genetic population structure among all sites (ϕ_ST_ = 0.025, p = 0.02), with significant differentiation among: (1) Kanamai, (2) southern Kenya, Tanzania, North and West Madagascar, (3) Southwest Madagascar and (4) East Madagascar (ϕ_CT_ = 0.035, p = 0.017). For *O*. *vulgaris* (n = 71, 482 bp), 15 haplotypes were identified, forming three haplogroups. A significant genetic population structure was found among all sites (ϕ_ST_ = 0.82, p ≤ 0.01). Based on pairwise ϕ_ST_-values and hierarchical AMOVAs, populations of *O*. *vulgaris* could be grouped as follows: (1) Brazil, (2) Madagascar and (3) all other sites. A significant increase in genetic distance with increasing geographic distance was found (Z = 232443, 81 r = 0.36, p = 0.039). These results indicate that for *O*. *cyanea* four regions should be considered as separate management units in the WIO. The very divergent haplogroups in *O*. *vulgaris* from Brazil and Madagascar might be evolving towards speciation and therefore should be considered as separate species in FAO statistics.

## Introduction

While the global market demand for octopus grows year after year, the supplies are becoming scarcer, and the prices are rising [1]. In 2017, China, Morocco and Spain were the largest exporters of octopus, capturing as much as 63% (164 000 tonnes) of the global production. In the same year, major import markets for octopus were located in the Republic of Korea, Japan and Italy [2]. A major limitation of FAO’s octopus catch statistics is the poor state of taxonomy, with only four species identified: *Octopus vulgaris* (Cuvier, 1797), *O*. *maya* (Voss & Solis Ramirez, 1966), *Eledone cirrhosa* (Lamarck, 1798) and *E*. *moschata* (Lamarck, 1798), while the remaining of the approximately 100 octopus species being landed is classified as ‘unidentified’ [3]. If the catch consists of more than one species, without this being noticed or recognised, the chance arises that the unidentified species are overexploited or even driven to extinction. Therefore, correct species identification of the catch is essential in order to get an idea of the potential impact of fisheries on abundance and viability of populations and to define adequate management and conservation measures [4]. The common octopus, *O*. *vulgaris*, was thought to be a cosmopolitan species, first described from the Mediterranean Sea and the Eastern North Atlantic and later reported in the subtropical and temperate East and West Atlantic Ocean, the Indian Ocean and West Pacific Ocean [5,6]. However, more recently it was suggested that populations previously treated as *O*. *vulgaris* comprise a group of morphologically similar but genetically distinct *vulgaris*-like species, the *O*. *vulgaris* species complex [7]. The species complex comprises *O*. *vulgaris sensu stricto* (s.s.), occurring in the Mediterranean and Eastern North Atlantic, and several *Octopus* “*vulgaris*” types that have been defined based on geographic distances, temperature boundaries, as well as the lack of plausible gene flow mechanisms [7]. *Octopus* “*vulgaris*” type I occurs in the tropical Western and Central Atlantic Ocean (the Caribbean and Gulf of Mexico). *Octopus* “*vulgaris*” type II inhabits the subtropical waters along the coast of Brazil in the Southwest Atlantic Ocean. *Octopus* “*vulgaris*” type III occurs in the temperate Southeast Atlantic, along the coast of South Africa and the South Indian Ocean. *Octopus* “*vulgaris*” type IV inhabits the temperate to subtropical waters of East Asia [7]. Over the past few years, several studies have identified the presence of cryptic species within the *O*. *vulgaris* species complex [4,8–12]. This highlights the necessity for correct species identification, since *O*. *vulgaris* is commercially the most valuable octopus species worldwide [3].

While octopus farming might be an option for the future [2], to date, octopus production relies on wild fisheries. Several fishing techniques are used for the capture of octopus, such as trawlers [13], pots and traps [14], harpoons [15] and spears [16]. In the Western Indian Ocean (WIO) octopus is mainly caught by women and children who walk at low spring tides on the exposed reef flats and probe octopus dens with spears, a fishing technique known as gleaning [16]. Due to the increasing market demand for cephalopods worldwide [1], these traditional African octopus fisheries have expanded quickly in recent decades and have shifted their focus from local and inland markets to international export [17]. Just as in other countries worldwide, landings of these artisanal African octopus fisheries are getting smaller [15] and concern over sustainability is raised [17, 18]. Besides *O*. *vulgaris*, the majority of the octopus catch in the WIO consists of the big blue octopus, *O*. *cyanea* (Gray, 1849) [15, 17–19]. The latter occurs on coral reefs in the Indo-Pacific Ocean, from East Africa and the Red Sea to Hawaii [7]. Both species are gonochoric and after mating the female lays up to 500,000 (*O*. *vulgaris*) or 700,000 eggs (*O*. *cyanea*) in a single clutch [7, 20]. The female takes care of the brood for approximately five weeks and dies shortly after hatching [21, 22]. In contrast to some members of the family Octopodidae, of which the hatchlings are quite large and immediately benthic, the majority of this family has small hatchlings that are planktonic and by convention are referred to as paralarvae [22]. The paralarvae of *O*. *cyanea* and *O*. *vulgaris* have a pelagic larval duration (PLD) of one to two months [21, 22] and during this larval stage dispersal between distant reefs can take place [20]. After reaching a critical size, the larvae begin the settlement process and become benthic [21, 22].

To maintain the sustainability of these socio-economic important octopus fisheries, management actions must be taken, such as the establishment of permanent Marine Protected Areas (MPAs) [19]. In these protected areas, extractive activities are limited or prohibited and are therefore effective in increasing the biomass of marine stocks, both within and outside the protected area [23]. When designing MPA networks, connectivity among populations through larval dispersal is a crucial factor, which should be taken into account, since connectivity determines gene flow as well as the ability to persist and recover from natural and anthropogenic stressors, such as overfishing [24]. Larval dispersal may be influenced by diverse factors, such as PLD, reproductive output, larval behaviour, direction and strength of ocean currents, geographic distance and oceanographic barriers [25]. Connectivity among populations can be determined using several genetic approaches. Mitochondrial DNA (mtDNA) is a widely used marker in population genetic studies since it possesses several advantageous characteristics. It is present in high numbers in the cell and is thereby easy to isolate [26]. Furthermore, mtDNA has a mosaic structure with fast evolving regions, resulting in a high variability, and more slowly evolving regions, allowing the use of universal primers. The latter makes mtDNA cheap and easy to amplify and sequence [26]. An example of a mtDNA marker is the cytochrome C oxidase subunit 1 (COI) gene, which has been applied as a DNA barcoding target gene in molluscs for species identification [27] and phylogeographic studies within a single species [28–31].

In order to obtain useful indications for the formulation of effective management plans, the present study investigated (1) the catch composition and diversity of Octopodidae in the Western Indian Ocean and (2) the connectivity and genetic structure of *O*. *cyanea* and *O*. *vulgaris* populations, using a fragment of the mitochondrial COI gene as marker. To the knowledge of the authors, this will be the first study dealing with population genetics of *O*. *cyanea*. Therefore, findings from this study are pioneering and can be used to further expand the knowledge base available for marine management decisions in the WIO.

## Materials and methods

### Study area

**Fig 1:**
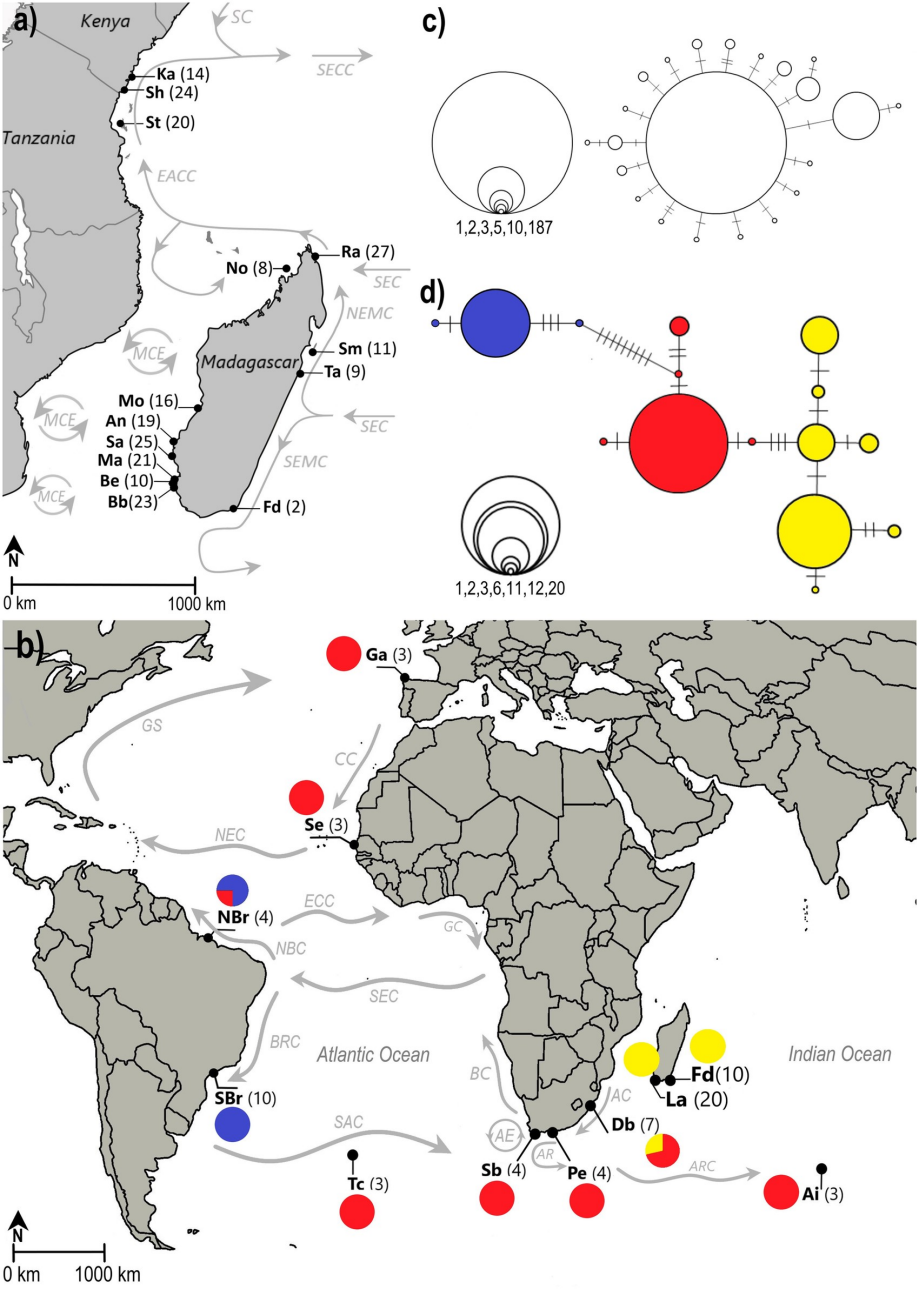
Maps showing sampling sites and haplotype networks for *Octopus cyanea* (a, c) and *O*. *vulgaris* (b, d). **(a)** Map of the Western Indian Ocean showing sampling sites for *O*. *cyanea* and prevailing currents during the Northeast Monsoon [32–34]. **(b)** Map of the Atlantic and Indian Ocean showing sampling sites for *O*. *vulgaris* and major currents [35,36]. Pie charts show the fractional contribution of the defined haplogroups in the haplotype network (see d) for each of the sample sites. Abbreviations: AC: Agulhas Current, AE: Agulhas Eddies, Ai: Amsterdam Island, An: Andavadoaka, AR: Agulhas Retroflection, ARC: Agulhas Return Current, Bb: Besambay, BC: Benguela Current, Be: Beheloke, BRC: Brazil Current, CC: Canary Current, Db: Durban, EACC: East African Coast Current, ECC: Equatorial Counter Current, Fd: Fort Dauphin, Ga: Galicia, GC: Guinea Current, GS: Gulf Stream, Ka: Kanamai, La: Lavanono, Ma: Maromena, MCE: Mozambique Channel Eddies, Mo: Morondava, NBC: North Brazil Current, NBr: North Brazil, NEC: North Equatorial Current, NEMC: Northeast Madagascar Current, No: Nosy Be, Pe: Port Elizabeth, Ra: Ramena, Sa: Salary, SAC: South Atlantic Current, Sb: Struisbaai, SBr: South Brazil, SC: Somali Current, Se: Senegal, SEC: South Equatorial Current, SECC: South Equatorial Counter Current, SEMC: Southeast Madagascar Current, Sh: Shimoni, Sm: Sainte Marie, St: Stone Town, Ta: Tamatave, Tc: Tristan da Cunha. Number of samples between brackets. **(c)** Network of 22 haplotypes from 229 individuals of *O*. *cyanea* and **(d)** network of 15 haplotypes from 71 individuals of *O*. *vulgaris*. The size of the circles is relative to the number of individuals represented by that haplotype. Hatches perpendicular to connection lines between circles indicate mutational steps.

For *O*. *cyanea*, the study focussed on the Western Indian Ocean. Because connectivity relies on dispersal of pelagic larvae, patterns are likely to be influenced by sea surface currents, moving larvae in a specific direction. In the WIO, the South Equatorial Current (SEC) flows from East to West across the Indian Ocean. Near the East coast of Madagascar, one component splits into the North-East Madagascar Current (NEMC) and South-East Madagascar Current (SEMC), while another one joins the NEMC at the Northern tip of Madagascar (Fig 1a). The latter continues West until it reaches the East African Coast, where it splits, creating on the one hand a number of southward propagating eddies on its way through the Mozambique Channel, and on the other hand the northward East African Coast Current (EACC), which converges with the seasonal (November-April) southward Somali Current (SC) at the northern coast of Kenya, joining into the South Equatorial Counter Current (SECC) (Fig 1a) [32–34].

### Sampling

Between 2014 and 2017, arm tips of 275 octopus individuals were collected in the WIO at landing sites and/or fish markets in Madagascar (Ramena, Nosy Be, Morondava, Andavadoaka, Salary, Maromena, Beheloke, Besambay, Lavanono, Fort Dauphin, Tamatave and Sainte Marie), Tanzania (Stone Town) and Kenya (Shimoni and Kanamai) (Fig 1a) (S1 Table). The samples were preserved in at least 95% ethanol and stored at 5 °C. A total of 41 sequences of *O*. *vulgaris* from South Africa, Brazil, Amsterdam Island, Tristan da Cunha, Senegal and Galicia were downloaded from the data portal of Barcode of Life Data Systems (BOLD Systems, http://v3.boldsystems.org/, last consulted on 11/07/2018) and GenBank (https://www.ncbi.nlm.nih.gov/genbank, last consulted on 11/07/2018) and included in the genetic population structure analysis of this study. The complete list of used COI sequences in this study can be found in S1 Table. Since the number of samples per sample site derived from the databases was often very low (n < 3), it was decided to combine sampling sites based on small geographic distances, the presence of favourable currents for connectivity and results found in the original studies. Sequences (n < 3) from sites that could not be combined or sequences that were too short (< 482 bp) were only used in the phylogenetic analysis.

### DNA extraction, PCR, and sequencing

A DNA extraction kit (E.Z.N.A. Tissue DNA Kit, Omega Bio-tek) was used for extracting DNA, following the manufacturer’s protocol. Success of DNA extraction was measured with a NanoDrop 2000 spectrophotometer (Thermo Scientific). The universal primers HCO2198 (5’-TAA ACT TCA GGG TGA CCA AAA AAT CA-3’) and LCO1490 (5’-GGT CAA CAA ATC ATA AAG ATA TTG G-3’), described by Folmer et al. [37], were used for the amplification of the COI gene fragment. PCR amplification was performed in a 25 μL reaction mixture containing 2 μL template DNA, 15.925 μL DNA free water, 1 μL (0.4 μM) of each forward and reverse primer, 2.5 μL (1 mM) PCR buffer, 0.5 μL (0.2 mM) dNTP, 0.875 μL (3.5 mM) MgCl_2_, 1 μl (10 mg/ml) BSA and 0.2 μL of (5 U/μL) Taq DNA polymerase, using a UNO96 VWR Thermal cycler. The following temperature profile was used: 5 min at 94 °C, followed by 40 cycles of 30 sec at 94 °C, 1 min at 51 °C and 1 min at 72 °C. Final extension was conducted at 72 °C for 10 min. After checking if the amplification was successful with gel electrophoresis on a 2% TBE agarose gel, fragment sequencing was carried out using an ABI 3730XL sequencer.

### Genetic diversity

The sequences were edited using the software CHROMASPRO (v. 1.5, Technelysium Ltd, Leicester, UK), i.e. the correct representation of nucleotides was checked based on the chromatogram peaks. DNA barcoding for species identification was performed using the Identification System (IDS) for COI from BOLD systems (BOLD Systems, http://v3.boldsystems.org/, last consulted on 11/07/2018). In addition, a Neighbour Joining (NJ) tree [38] including bootstrap analysis (1000 replications) [39] was constructed using the software MEGA 7 (v. 7.0) [40]. For the latter, a few reference sequences of each identified species were downloaded from BOLD Systems (S1 Table).

Multiple and pairwise alignment of the sequences was carried out using Clustal W [41] as implemented in the software MEGA 7 and sequences were trimmed to the shortest common sequence length. The same program was further used to translate COI sequences into amino-acid sequences as quality control to exclude sequencing errors and/or to verify if functional mitochondrial DNA sequences were obtained and not nuclear pseudogenes. The online service FaBox DNA Collapser [42] (http://users-birc.au.dk/biopv/php-/fabox/dnacollapser.php#, last consulted on 12/07/2018) was used to reduce the sequence dataset to haplotypes. Nucleotide and haplotype diversities for all populations were calculated according to Nei [43] with the program Arlequin (v. 3.5) [44].

### Historical demography

Unless otherwise stated, all following analyses were carried out with Arlequin. To insure suitability for population genetic analyses, the null hypothesis of neutral evolution of the marker was evaluated on the basis of Tajima’s D [45] and Fu ‘s F_S_ [46] tests, which also allows the detection of a recent population expansion or bottleneck. In order to discriminate between selective pressure and population expansion and investigate the historical demography, mismatch distribution analysis was performed [47] and the sum of square deviation [48] and Harpending’s raggedness index [49] were calculated, thereby testing the model of sudden population expansion [50].

### Genetic population structure and connectivity

An analysis of molecular variance was performed to investigate the overall genetic population structure in the dataset (ϕ_ST_) (AMOVA; [51]) and pairwise population differentiations (pairwise ϕ_ST_) were determined. The pairwise ϕ_ST_ P-values were adjusted using Holm’s sequential Bonferroni correction [52]. Groups for hierarchical AMOVA were chosen to test for genetic population structure across potential oceanographic barriers. Geographic distances between sample sites were measured as the shortest connection via marine pathways using Google Earth. The Isolation by Distance Web Service ([53]; http://ibdws.sdsu.edu, last consulted on 14/07/2018) was then used to assess the correlation between geographic and genetic distances (pairwise ϕ_ST_ -values) of sampled populations by applying a Mantel test [54]. A haplotype network was drawn based on the Minimum Spanning Tree information provided by Arlequin in order to examine the relationship among haplotypes.

## Results

**Fig 2:**
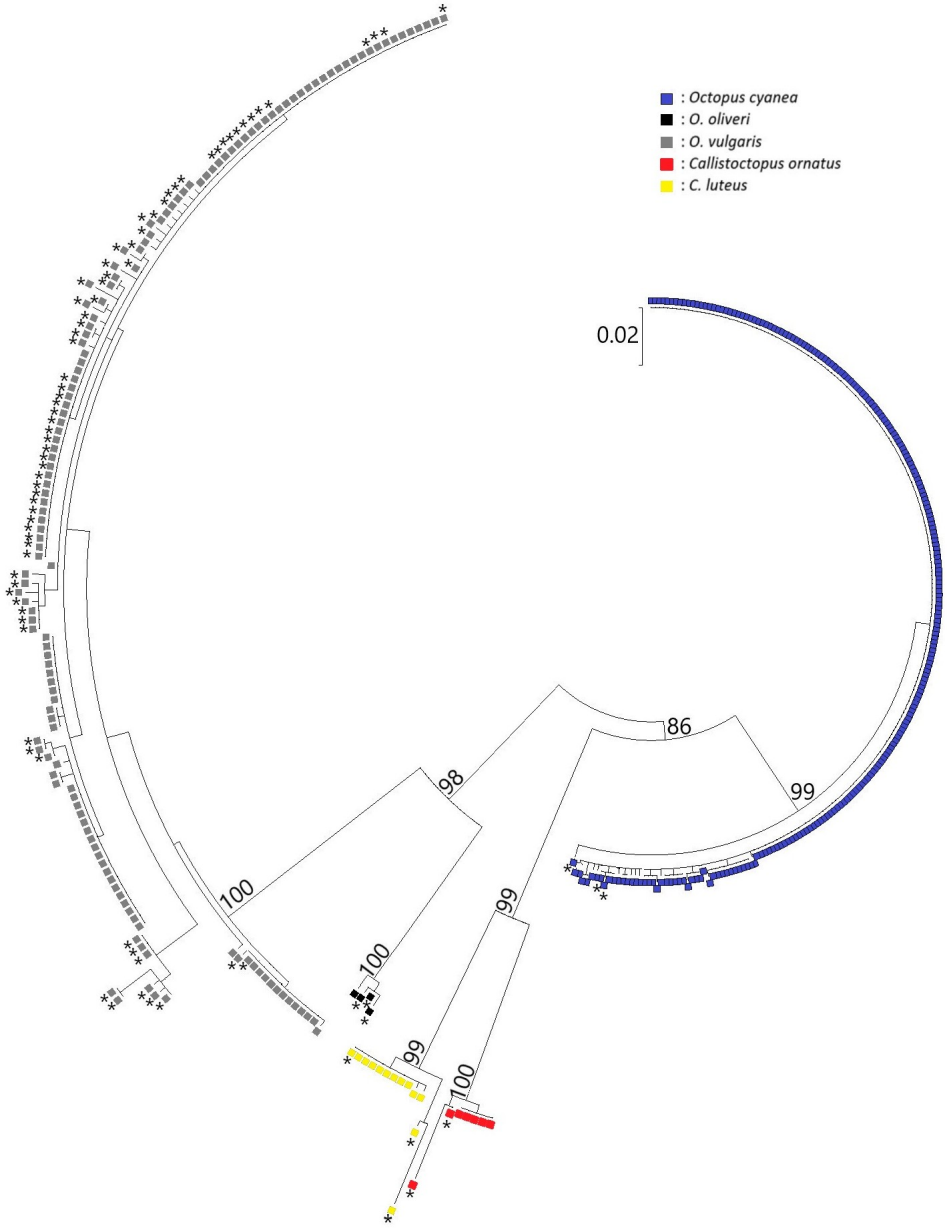
Neighbour-Joining (NJ) tree of all octopus samples and reference sequences downloaded from BOLD Systems (*). Bootstrap values are indicated at the major branches. Individuals of the same species are clustered together.

COI sequences of 275 octopus individuals, from 15 locations, were successfully amplified and deposited in Genbank (accession numbers: MK593176 –MK593450). Of those 275 samples, 229 were identified as *O*. *cyanea*, 30 as *O*. *vulgaris*, ten as *Callistoctopus luteus*, five as *C*. *ornatus* and one as *O*. *oliveri* (Fig 2, S1 Table). At all sample sites, all collected individuals were identified as *O*. *cyanea*, except Lavanono (La, n = 21), of which 20 samples were *O*. *vulgaris* and one sample being *O*. *oliveri*, and Fort Dauphin (Fd, n = 27), of which two samples were *O*. *cyanea*, ten samples were *O*. *vulgaris*, ten samples were *C*. *luteus* and five samples were *C*. *ornatus*.

### Octopus cyanea

#### Genetic diversity

Based on an alignment of 229 *O*. *cyanea* COI sequences with a length of 563 base pairs, 22 haplotypes were defined. Haplotype diversity (*h*) within each population ranged from 0 in Nosy Be (No) and Morondava (Mo) to 0.71 in Beheloke (Be). Nucleotide diversity (*π*) ranged from 0 in No and Mo to 0.15 in Be (Table 1).

**Table 1:** Measures of genetic diversity, tests for neutrality and mismatch distributions for *Octopus cyanea* and *O*. *vulgaris*.

	Sample site	Country	Code	n	*N*_*hap*_	Genetic Diversity		Neutrality test		Mismatch distribution	
						h	π (%)	Tajima’s D	Fu’s Fs	SSD	HRI
***O*. *cyanea***	Kanamai	KE	Ka	14	4	0.49 ± 0.15	0.12 ± 0.11	-0.89	-1.29	0.000	0.083
	Shimoni	KE	Sh	24	2	0.08 ± 0.07	0.03 ± 0.05	-1.51**	-0.19	0.010	0.854
	Stone Town	TZ	St	20	6	0.45 ± 0.14	0.09 ± 0.09	-1.97**	-4.62**	0.006	0.140
	Ramena	MG	Ra	27	5	0.28 ± 0.11	0.07 ± 0.07	-2.00**	-3.50**	0.000	0.294
	Nosy Be	MG	No	8	1	0.00 ± 0.00	0.00 ± 0.00	N\A	N\A	N\A	N\A
	Morondava	MG	Mo	16	1	0.00 ± 0.00	0.00 ± 0.00	N\A	N\A	N\A	N\A
	Andavadoaka	MG	An	19	6	0.47 ± 0.14	0.11 ± 0.10	-2.05**	-3.87	0.000	0.096
	Salary	MG	Sa	25	4	0.23 ± 0.11	0.06 ± 0.07	-1.50	-2.44**	0.002	0.395
	Beheloke	MG	Be	10	4	0.71 ± 0.12	0.15 ± 0.13	-0.66	-1.18	0.040	0.260
	Besambay	MG	Bb	23	4	0.32 ± 0.12	0.06 ± 0.07	-1.48	-2.32**	0.002	0.223
	Maromena	MG	Ma	21	3	0.34 ± 0.12	0.08 ± 0.08	-1.22	-0.39	0.000	0.206
	Fort Dauphin	MG	Fd	2	2	N\A	N\A	N\A	N\A	N\A	N\A
	Tamatave	MG	Ta	9	3	0.42 ± 0.19	0.08 ± 0.09	-1.36	-1.08*	0.009	0.169
	Sainte Marie	MG	Sm	11	5	0.62 ± 0.16	0.13 ± 0.12	-1.71*	-2.91**	0.024	0.188
***O***. ***vulgaris***	North Brazil	BR	NBr	4	3	0.83 ± 0.22	1.30 ± 0.94	-0.07	1.92	0.201	0.528
	South Brazil	BR	SBr	10	2	0.20 ± 0.15	0.04 ± 0.07	-1.11	-0.34	0.331	0.400
	Galicia	SP	Ga	3	1	0.00 ± 0.00	0.00 ± 0.00	N\A	N\A	N\A	N\A
	Senegal	SE	Se	3	1	0.00 ± 0.00	0.00 ± 0.00	N\A	N\A	N\A	N\A
	Tristan da Cunha	GB	Tc	3	1	0.00 ± 0.00	0.00 ± 0.00	N\A	N\A	N\A	N\A
	Struisbaai	SA	Sb	4	3	0.83 ± 0.22	0.21 ± 0.21	-0.71	-0.89	0.113	0.528
	Port Elizabeth	SA	Pe	4	1	0.00 ± 0.00	0.00 ± 0.00	N\A	N\A	N\A	N\A
	Durban	SA	Db	7	3	0.52 ± 0.21	0.55 ± 0.38	0.45	1.93	0.390**	0.299
	Lavanono	MG	La	20	5	0.80 ± 0.05	0.25 ± 0.19	1.12	-0.65	0.019	0.153
	Fort Dauphin	MG	Fd	10	4	0.73 ± 0.12	0.29 ± 0.22	-0.10	-0.21	0.013	0.068
	Amsterdam island	FR	Ai	3	1	0.00 ± 0.00	0.00 ± 0.00	N\A	N\A	N\A	N\A

Abbreviations: BR = Brazil; FR = France; GB = Great Britain; KE = Kenya; MG = Madagascar; SA = South Africa; SE = Senegal; SP = Spain; TZ = Tanzania; n = sample size; N_hap_ = haplotype number; *h* = haplotype diversity (± SD); *π* = nucleotide diversity (± SD); SSD = sum of square deviation; HRI = Harpending’s raggedness index.

Significance levels:

*0.05 > p > 0.01;

**p ≤ 0.01;

all other values not significant, p ≥ 0.05. N\A: not applicable.

#### Historical demography

The null hypothesis of neutral evolution of the COI marker was rejected for five sample sites based on Tajima’s D-test, whereas the results of Fu’s Fs test rejected the null hypothesis for six sites (Table 1). These significant outcomes could indicate selection or departures from population equilibrium (e.g. population expansion). The latter is supported by non-significant values for SSD and HRI for all sample sites, indicating no significant deviation from a model of sudden demographic expansion.

#### Genetic population structure and connectivity

The evolutionary relationships among 22 *O*. *cyanea* haplotypes are presented in a haplotype network, showing one haplogroup (Fig 1c). AMOVA revealed a very low but significant overall genetic structure (ϕ_ST_ = 0.025, p = 0.02), rejecting the null hypothesis of panmixia. Pairwise ϕ_ST_-values between sample sites showed homogeneity among most of them, with significant differentiation visible in only two out of the 91 pairwise population comparisons, after applying sequential Bonferroni correction (S2 Table). A hierarchical AMOVA was carried out, with all tested groupings being significant (Table 2). However, grouping (7) had a slightly higher and significant fixation index: (1) Kanamai (Ka), (2) southern Kenya, Tanzania, North Madagascar, West Madagascar (Sh, St, Ra, No, Mo), (3) Southwest Madagascar (An, Sa, Be, Bb, Ma) and (4) East Madagascar (Fd, Sm, Ta) (ϕ_CT_ = 0.035, p = 0.017). Analysis of isolation by distance revealed no significant increase in genetic distance with increasing geographic distance (Z = 6847.87, r = 0.08, p = 0.25).

**Table 2:** Hierarchical AMOVA of O*ctopus cyanea* populations in the Western Indian Ocean.

	Grouped populations	Barrier	ϕ_CT_
1	East Africa vs Madagascar	1	0.023*
	(Ka, Sh, St) vs (Ra, No, Mo, An, Sa, Be, Bb, Ma, Fd, Ta, Sm)		
2	East Africa vs East Madagascar vs West Madagascar	1,2,3	0.026*
	(Ka, Sh, St) vs (No, Mo, An, Sa, Be, Bb, Ma) vs (Ra, Sm, Ta, Fd)		
3	Kenya vs Tanzania vs West Madagascar vs East Madagascar	1,2,3,4	0.024*
	(Ka, Sh) vs (St) vs (No, Mo, An, Sa, Be, Bb, Ma) vs (Ra, Sm, Ta, Fd)		
4	East Africa vs West Madagascar vs Southwest Madagascar vs East Madagascar	1,2,3,5	0.026*
	(Ka, Sh, St) vs (No, Mo, An, Sa) vs (Be, Bb, Ma) vs (Ra, Sm, Ta, Fd)		
5	East Africa and East Madagascar vs West Madagascar	2,6	0.027*
	(Ka, Sh, St, Ra,Fd, Ta, Sm) vs (No, Mo, An, Sa, Be, Bb, Ma)		
6	East Africa, North and West Madagascar vs Southwest Madagascar vs East Madagascar	2,5,7	0.033*
	(Ka, Sh, St, No, Ra, Mo) vs (An, Sa, Be, Bb, Ma) vs (Sm, Ta, Fd)		
7	Kanamai vs South Kenya & Tanzania & N Madagascar & W Madagascar vs SW Madagascar vs E Madagascar	2,5,7,8	0.035*
	(Ka) vs (Sh, St, No, Ra, Mo) vs (An, Sa, Be, Bb, Ma) vs (Sm, Ta, Fd)		

Potential barriers to gene flow include: (1) open water between East Africa and Madagascar, (2) Retroflection of the SEMC, (3) adapted from Ramanantsoa et al., 2018, (4) isolation of island populations, (5) Mozambique Channel Eddies, (6) split of the SEC at east coast of Africa, (7) split of the SEC at East coast of Madagascar, (8) convergence of the EACC and SC. For abbreviations of samples sites see Table 1.

Significance levels:

*0.05 > p > 0.01;

**p ≤ 0.01;

### Octopus vulgaris

#### Genetic diversity

Based on an alignment of 71 *O*. *vulgaris* COI sequences with a length of 482 base pairs, 15 haplotypes were defined. Haplotype diversity (*h*) within each population ranged from 0 in Ga, Se, Tc, Pe, and AI to 0.83 in NBr and Sb. Nucleotide diversity (*π*) ranged from 0 in Ga, Se, Tc, Pe, and AI to 0.013 in NBr (Table 1).

#### Historical demography

The null hypothesis of neutral evolution of the COI marker was accepted for all sample sites based on both Tajima’s D-test and Fu’s Fs test (Table 1). Departures from population equilibrium are supported by non-significant values for SSD (except Db) and HRI for all sample sites, indicating no significant deviation from a model of sudden demographic expansion.

#### Genetic population structure and connectivity

The evolutionary relationships among 15 *O*. *vulgaris* haplotypes are presented in a haplotype network, showing three haplogroups (Fig 1d). The distribution of haplogroups is shown in Fig 1(b). Haplotypes from the blue haplogroup were only present at Brazilian sites, while the yellow haplogroup was only present at sites in Madagascar and in Db. AMOVA showed a significant genetic structure in the overall sample (ϕ_ST_ = 0.82, p ≤ 0.01), rejecting the hypothesis of panmixia. The genetic differentiation between Brazilian, Malagasy and all other sites was confirmed by very high and significant pairwise ϕ_ST_-values (Table 3). Based on oceanography, hierarchical AMOVAs were carried out, with all tested groupings being significant (Table 4). However, grouping (1) had a slightly higher and highly significant fixation index: Brazil vs all other sites (ϕ_CT_ = 0.902, p ≤ 0.01). Since the population structure seems to be mainly driven by the samples from Brazil, with the blue haplogroup only occurring in that region and a minimum of nine mutation steps between the red and blue haplogroup (Fig 1d), the analysis was repeated without the samples from Brazil. However, the AMOVA still detected a high and significant level of genetic population structure (ϕ_ST_ = 0.69, p ≤ 0.01). Other hierarchical AMOVAs were carried out, with all tested groupings being significant (Table 5). However, grouping (1) had a slightly higher and highly significant fixation index: Madagascar vs all other sites (ϕ_CT_ = 0.773, p ≤ 0.01). Furthermore, analysis of isolation by distance revealed a significant increase in genetic distance with increasing geographic distance (Z = 232443, 81 r = 0.36, p = 0.039).

**Table 3:** Pairwise ϕ_ST_ values between populations of *Octopus vulgaris*.

	**NBr**	**SBr**	**Ga**	**Se**	**Tc**	**Sb**	**Pe**	**Db**	**La**	**Fd**	**AI**
**NBr**	0.00										
**SBr**	0.36	0.00									
**Ga**	0.63	0.99**	0.00								
**Se**	0.59	0.99	1.00	0.00							
**Tc**	0.59	0.99**	1.00	0.00	0.00						
**Sb**	0.61	0.97**	0.83	0.00	0.00	0.00					
**Pe**	0.65**	0.99**	1.00	0.00	0.00	0.00	0.00				
**Db**	0.59	0.91**	0.54	0.00	0.00	0.00	0.04	0.00			
**La**	0.81**	0.94**	0.83**	0.80**	0.80**	0.77**	0.81**	0.58**	0.00		
**Fd**	0.75**	0.94**	0.83**	0.79	0.79**	0.76**	0.81**	0.53**	0.11	0.00	
**AI**	0.59	0.99**	1.00	0.00	0.00	0.00	0.00	0.00	0.80**	0.79	0.00

For abbreviations of samples sites see Table 1. Negative values have been replaced with zero.

Significance level:

**p ≤ 0.01;

all other values not significant after sequential Bonferroni correction (p ≥ 0.05).

**Table 4:** Hierarchical AMOVA of *Octopus vulgaris* populations.

	Grouped populations	Barrier	ϕ_CT_
1	Brazil vs all others sites	1	0.902**
	(NBr, SBr) vs (Ga, Se, TC, Sb, Pe, Db, La, Fd, AI)		
2	Atlantic vs Indian Ocean	2	0.856**
	(NBr, SBr, Ga, Se, TC) vs (Sb, Pe, Db, La, Fd, AI)		
3	West Atlantic vs East Atlantic vs Western Indian Ocean vs Southern Indian Ocean	1,2,3	0.865**
	(NBr, Sbr) vs (Ga, Se, TC) vs (Sb, Pe, Db, La, Fd) vs (AI)		
4	Brazil vs Atlantic Ocean and Southern South Africa vs Indian Ocean	1,2	0.861**
	(NBr, SBr) vs (Ga, Se, TC, Sb, Pe) vs (Db, La, Fd, AI)		
5	North Brazil vs South Brazil vs Atlantic Ocean and South Africa vs Indian Ocean	1,2,4	0.859**
	(NBr) vs (SBr) vs (Ga, Se, TC, Sb, Pe) vs (Db, La, Fd, AI)		
6	Brazil vs Atl. Ocean vs South Africa vs Madagascar vs Southern Indian Ocean	1,2,3,5	0.843**
	(NBr, Sbr) vs (Ga, Se, TC) vs (Sb, Pe, Db) vs (La, Fd) vs (AI)		
7	Brazil vs NE Atl. Ocean vs east Atl. Ocean and South Africa vs Madagascar vs Southern Indian Ocean	1,3,5,6	0.850**
	(NBr, SBr) vs (Ga) vs (Se, TC, Sb, Pe, Db) vs (La, Fd) vs (AI)		
8	Brazil vs NE Atl. Ocean vs east Atl. Ocean vs South Africa vs Madagascar vs Southern Indian Ocean	1,2,3,5,6	0.843**
	(NBr, SBr) vs (Ga) vs (Se, TC) vs (Sb, Pe, Db) vs (La, Fd) vs (AI)		
9	Brazil vs NE Atl. Ocean vs E Atl. Ocean vs central Atl. Ocean vs S. Africa vs Madagascar vs S.Indian Ocean	1,2,3,5,6,7	0.842**
	(NBr, SBr) vs (Ga) vs (Se) vs (TC) vs (Sb, Pe, Db) vs (La, Fd) vs (AI)		

Potential oceanographic barriers include: (1) open ocean between West and East Atlantic Ocean, (2) Agulhas Retroflection, (3) open ocean between WIO and Southern Indian Ocean, (4) split of the SEC into NBC and BRC, (5) Mozambique Channel Eddies, (6) temperature boundary between temperate and subtropical water, (7) South Atlantic Current. For abbreviations of samples sites see Table 1.

Significance level:

**P ≤ 0.01.

**Table 5:** Hierarchical AMOVA of *Octopus vulgaris* populations without populations from Brazil.

	Grouped populations	Barrier	ϕ_CT_
1	All other sites vs Madagascar	1	0.773**
	(Ga, Se, TC, Sb, Pe, Db, AI) vs (La, Fd)		
2	Atlantic Ocean and South Africa vs Madagascar vs Southern Indian Ocean	1,2	0.758**
	(Ga, Se, TC, Sb, Pe, Db) vs (La, Fd) vs (AI)		
3	Northern Atlantic Ocean vs Eastern Atlantic Ocean and South Africa vs Madagascar vs Southern Indian Ocean	1,2,3	0.756**
	(Ga) vs (Se, TC, Sb, Pe, Db) vs (La, Fd) vs (AI)		
4	Northern Atlantic Ocean vs Eastern Atlantic Ocean vs South Africa vs Madagascar vs Southern Indian Ocean	1,2,3,4	0.739**
	(Ga) vs (Se, TC) vs (Sb, Pe, Db) vs (La, Fd) vs (AI)		

Potential oceanographic barriers include: (1) Mozambique Channel Eddies, (2) open ocean between WIO and Southern Indian Ocean, (3) temperature boundary between temperate and subtropical water, (4) Agulhas Retroflection. For abbreviations of samples sites see Table 1.

Significance level:

**P ≤ 0.01.

## Discussion

### Biodiversity in the Western Indian Ocean

While *O*. *cyanea*, *O*. *vulgaris* and *C*. *ornatus* are known to occur in the waters of the Western Indian Ocean [7, 15, 17–19, 55], this is the first record of *C*. *luteus* and *O*. *oliveri* in these waters. This is a substantial range expansion because until now, *O*. *oliveri* and *C*. *luteus* are only known from the Western Pacific Ocean [7, 27, 55–56], with one individual of *C*. *luteus* being found in India (Accession number KF489434; unpubl. data). Therefore, the actual distribution range (e.g. in World Register of Marine Species (WoRMS) database) should be updated.

At all sample sites all collected individuals were identified as *O*. *cyanea*, except in the two most southern located sites in Madagascar, Lavanono (La) and Fort Dauphin (Fd). In the former, samples consisted of *O*. *vulgaris* and *O*. *oliveri*, with *O*. *cyanea* not represented, while in the later samples consisted of *O*. *vulgaris*, *C*. *luteus*, *C*. *ornatus* and only a small fraction of *O*. *cyanea*. A possible explanation could be the difference in Sea Surface Temperature (SST). All sample sites are located in the tropics, with SST ranging between 26.0 °C and 28.2 °C. However, La and Fd are located more south, in sub-tropical waters, with SST below 25.0 °C [57]. Egg laying and rearing under aquarium conditions was done at 18.2 °C—20.0 °C for *C*. *luteus* [58], at 21.2 °C for *O*. *vulgaris* [22], at an average of 25.1 °C for *O*. *oliveri* [59] and at an average of 25.5 °C for *O*. *cyanea*, with most rapid development at temperatures above 27.1 °C [21]. For *C*. *ornatus* no such data was available. From these experiments it seems that, even if ranges might overlap [15, 60–61], the optimal SST might be too low for *O*. *cyanea* in the southern located sites, while temperature might be too warm for the other octopus species in more northern located sites. Another explanation could be the differences in fishing time period. Traditionally, women and children catch octopus by gleaning at low spring tides on the exposed reef flats and probe octopus dens with spears [16]. Depending on when low spring tides occur, different species might be caught, since *O*. *cyanea* is active during the day [62], while *C*. *ornatus* [62], *C*. *luteus* [61], *O*. *oliveri* [63] and *O*. *vulgaris* [64] are nocturnal species. Also, stronger current and higher wave activity in South Madagascar [65] might be a plausible explanation for the observed distribution pattern. However, since samples were collected from February to October over several years, with southern locations sampled in May, a seasonal effect can be excluded.

### Genetic diversity and historical demography

All populations of *O*. *cyanea* and *O*. *vulgaris* showed a moderate level of haplotype diversity (*h*) and a low level of nucleotide diversity (*π*). This pattern has also been reported in other studies in the WIO [30, 66]. The high *h* and low *π* might indicate a genetic bottleneck that has caused stochastic extinction of most haplotypes, followed by population expansion [67]. This is congruent with the results of Tajima’s D-test, Fu’s Fs test, the mismatch distribution analysis and Rogers’ test for sudden population expansion, that suggest demographic growth. This finding is similar to the population expansion reported in the WIO for the African giant mud crab *Scylla serrata* [66], the skunk clownfish *Amphiprion akallopisos* [68] and the mangrove whelk *Terebralia palustris* [30].

### Genetic population structure and connectivity

#### Octopus cyanea

The results from this study indicate that the WIO population of *O*. *cyanea* is characterised by a dominant haplotype that is present at all sample sites (Fig 1c). The haplotype network has a star-like structure, with a central haplotype surrounded by several smaller ones that show only little base pair differences. This might be an indication of recent population expansion from a small number of founders, supporting our previous findings. Even though all sites share the most common haplotype and the network suggests panmixia, a very weak but significant genetic population structure was found across all sample sites in the WIO (ϕ_ST_ = 0.025, P = 0.02). Based on a hierarchical AMOVA, restricted gene flow could be detected between *O*. *cyanea* populations from (1) Kanamai (Ka), (2) southern Kenya (Sh), Tanzania (St), North and West Madagascar (Ra, No, Mo), (3) Southwest Madagascar (An, Sa, Ma, Be, Bb) and (4) East Madagascar (Sm, Ta, Fd). Similar findings have been reported for *Terebralia palustris*, for which restricted gene flow was detected between (1) northern Kenya, (2) Kenya, Tanzania and western Madagascar, (3) northeastern Madagascar and (4) southern Madagascar [30]. For the giant mud crab *Scylla serrata* a significant genetic divergence was found between sites in eastern Madagascar and sites on the West coast of Madagascar, mainland East Africa, as well as the Seychelles [66]. At a smaller scale, a weak genetic structure was detected among populations of *Acropora tenuis* in Tanzania and Kenya [69]. On the contrary, some studies in the WIO regarding invertebrates found high connectivity and no genetic structure [70, 71]. This lack of genetic population structure could be attributed to the absence of samples from Madagascar in both studies.

No significant correlation was found between genetic differentiation and geographical distance. Therefore, the main factor that restricts gene flow and thus dispersal seems to be the direction and strength of ocean currents. During the Northeast Monsoon, the East African Coast Current (EACC) and the Somali Current (SC) converge into the eastward South Equatorial Counter Current (SECC) [32], which may potentially prevent dispersal from southern locations to the northernmost sample site Kanamai (Ka). The South Equatorial Current (SEC) may transport larvae from North Madagascar to Tanzania and Kenya, by splitting of the EACC, and to the northwest coast of Madagascar by the southward split of the SEC. In addition, islands like the Comoros, Mayotte, and Îles Glorieuse could serve as stepping-stones between western Madagascar and East Africa [30]. An experiment with satellite-tracked drifters demonstrated that eddies in the Mozambique Channel (MCE) are capable of trapping drifting objects for periods of several weeks to months [33]. Differentiation between southwestern sites and other localities may therefore be due to trapping, isolation and retention of the larvae inside southward propagating eddies. The splitting of the SEC into the southward Southeast Madagascar Current (SEMC) and the northward Northeast Madagascar Current (NEMC) at the East coast of Madagascar might be a reason for restricted gene flow between sites in Northeast Madagascar and southern sites. A similar pattern was found for the skunk clownfish, *Amphiprion akallopisos*, for which the population from Sm was significantly differentiated from the other WIO populations [68]. The southernmost location in Madagascar (Fd) is very likely under the influence of the SEMC, what might prevent exchange and gene flow with sample sites at the West coast of Madagascar or African mainland. Such a separation was also observed in *Terebralia palustris* [30]. However, a recent paper suggests that mtDNA sequence data are often not suitable to test for IBD because mtDNA often fails to reliably detect such pattern when other markers, like microsatellites, can. This failure of mtDNA seems to be the result of a reduction in genetic diversity due to selection, which can obscure spatial genetic differentiation [72].

The results of this study suggest there is high genetic exchange of *O*. *cyanea* in the WIO region due to the long-distance dispersal capacity of *O*. *cyanea* paralarvae. However, currents are one of the main factors responsible for restricted gene flow to a certain extent between the central part (Kenya, Tanzania, north- and western Madagascar), Kanamai (convergence of EACC and SC into SECC), eastern Madagascar (NEMC and SEMC) and southwestern Madagascar (EACC retroflection and MCE). It is important to remark that the small number of samples for certain sites may have influenced the results that were found. Furthermore, we hypothesise that the contribution of adult migration to population connectivity is negligible. Adult *O*. *cyanea* may use one den for three to five weeks [62, 73]. Even though no records are available of the average distance between old and new dens for *O*. *cyanea*, it is known for other octopus species that this distance is around 15 m [74]. If these distances would also apply for *O*. *cyanea* then the species appears to be relatively resident and migration of adult *O*. *cyanea* is negligible. However, research needs to be done to confirm this hypothesis.

#### Octopus vulgaris

A strong and significant population structure (ϕ_ST_ = 0.82, p ≤ 0.01), was found among all sample sites. Based on very high and significant pairwise ϕ_ST_-values and hierarchical AMOVA, restricted gene flow could be detected between populations from Brazil, Madagascar and all other sampling sites. The strong genetic differentiation of the Brazilian and Malagasy sites can be attributed to the exclusive occurrence of haplotypes from the blue (Brazil) and yellow (Madagascar) haplogroup (Fig 1b and 1d). In Durban (Db), the yellow haplogroup is also present. Another study found that specimens of *O*. *vulgaris* from Db were genetically more different from other South African sites than specimens from Senegal or Tristan Da Cuhna in the southern Atlantic Ocean [60]. The authors suggested that the dominance of one haplogroup along the coast of South Africa, with another genetic lineage so far only found in Durban, might be the result of a recent introduction of the latter, in which case identical haplotypes should be found in other regions [60]. Our study can confirm that one of the two haplotypes belonging to the yellow haplogroup in Durban is also present in Madagascar (La and Fd). This might indicate that paralarvae of *O*. *vulgaris* may be transported from Madagascar to Durban, in which case the latter might act in the future as a stepping stone for dispersal of paralarvae between Madagascar and South Africa. An experiment with satellite-tracked drifters in the Mozambique Channel showed that cross-channel transport between Madagascar and Mozambique was possible, with time spans of 19–30 days [33]. If the yellow haplogroup has its origin in Madagascar, this might be the result of a colonisation event of Madagascar, after which the Malagasy subpopulation started to evolve towards insular speciation (followed by possible dispersal to Durban, African mainland), as can be observed in many of the terrestrial fauna and flora on the island [75]. However, another possibility is that the ancestral haplotype of the yellow haplogroup has its origin in Mozambique and that larvae from that region are transported south to Madagascar and Durban by Mozambique Channel Eddies. This was found for *T*. *palustris*, in which southern Mozambique was postulated as potential origin of the populations elsewhere in the WIO [30]. In this case, gene flow between Madagascar and South Africa might not take place. Restricted gene flow between the southern African mainland and Madagascar is found by other studies. For the spiny lobster *Palinurus delagoae*, a genetic break was found between populations from South Africa and Walters Shoals, a submerged seamount on the Madagascar Ridge [76]. For the mangrove whelk *Terebralia palustris*, significant differentiation was found between southern Madagascar and southern Mozambique [30]. Giant mud crab (*Scylla serrata*) populations from South Africa and the West Coast of Madagascar did not show significant genetic differentiation among each other, but a significant genetic break was observed with populations from East Madagascar [66]. Due to low sample size in the South African localities and the limited number of sites investigated, further sampling and research needs to be conducted in order to confirm either of both hypothesis. Anyhow, the uniqueness of populations from the WIO compared with populations elsewhere in the Indian Ocean was also found for other species such as the skunk clownfish [68] and the mud crab *Scylla serrata* [77].

The exclusive presence of the blue haplogroup in Brazilian populations, together with very high and significant pairwise ϕ_ST_-values and the hierarchical AMOVA, indicate very high differentiation with individuals from the other sample sites. This is congruent with previous studies that found morphological [11, 78] and molecular evidence [8, 11] that *O*. *vulgaris* from Brazil might be a distinct species [11]. Furthermore, other studies found significant differentiation with microsatellite markers along the southern Brazilian coast [79].

The isolation-by-distance analysis revealed a significant correlation between genetic and geographical distances. Therefore, geographic distance seems to be one of the main factors that restricts gene flow and hence dispersal. Paralarvae of *O*. *vulgaris* settle and become benthic between day 47 and 54 after hatching [22]. However, time of settlement is independent of age, but related to reaching a critical size (173 mg and 7.5 mm mantle length, at an average temperature of 21.2 °C) and the duration of the planktonic life stage is temperature dependent [22]. Another study recorded a paralarvae duration of 22–30 days at 24.3–26.9 °C [80]. The species has a higher growth rate at high water temperatures, due to an increased food intake rate [81]. Following that reasoning, paralarvae have a higher dispersal distance in temperate waters than in (sub)tropical waters. The non-significant pairwise ϕ_ST_-values and the presence of the red haplotypes in the East Atlantic Ocean, South Africa and the Southern Indian Ocean suggest high connectivity and are not in line with the results of the isolation-by-distance analysis. The most plausible explanation for these results is that due to the low sampling size other haplotypes are overlooked and thereby gene flow is overestimated. Another explanation might be that there is high dispersal of *O*. *vulgaris* paralarvae among the East Atlantic Ocean, South Africa and Amsterdam Island in the Southern Indian Ocean. The Canary and Benguela Currents may transport paralarvae in the East Atlantic Ocean, which are expected to have high dispersal capacities reasoned by the cold character of the currents [36, 82]. Water between the WIO and the Atlantic Ocean can be exchanged through the shedding of Agulhas Eddies (AE) from the Agulhas Retroflection (AR). Paralarvae may then be transported towards Amsterdam Island by the Agulhas Return Current (ARC) [36, 83]. Even though genetic differentiation was found for *O*. *vulgaris* along the Senegalese and Mauritanian coast [84], such high connectivity levels between different oceans were observed for sea urchins [85] and bigscale soldierfish [86]. Further sampling and research need to be conducted to confirm either hypothesis.

The complete analysis was also conducted with sequences of 462 bp, which included samples from Greece (Accession numbers: JX500627—JX500639) and more samples of Galicia (Accession numbers: JX500655—JX50065577) (S1 Table). Since we lost 4% of the genetic data compared with the present analysis (482 bp) it was decided to use less but longer sequences. Samples from Greece belonged to the red haplogroup with most sequences belonging to the dominant/ancestral haplotype. The samples from Galicia were also part of the red haplogroup, but most sequences belonged to a haplotype that differed three mutation steps from the dominant/ancestral haplotype.

### Future research

Over the past couple of years, concern was raised about the exclusive use of mtDNA markers, since they only reflect one locus, only showing the maternal genetic history [87] and they might be under selection in many animal species [88]. To be sure that the observed genetic structure patterns that were found with COI are not influenced by these shortcomings, nuclear loci should be analysed additionally in the future [87]. By comparing the results of mtDNA and nuclear markers such as microsatellites, the suitability of mtDNA can be validated and additional information might be obtained [66, 68].

## Conclusions

More species of the family Octopodidae occur in the WIO than previously thought. Furthermore, this study revealed high levels of connectivity among *O*. *cyanea* populations in the WIO, with low but significant differentiation between the central part (Kenya, Tanzania, north- and western Madagascar), Kanamai, eastern Madagascar and southwestern Madagascar. For *O*. *vulgaris* highly restricted gene flow could be detected between populations from Brazil, Madagascar and all other sampling sites due to the large geographical distance. Therefore, this study suggests that four separate management units should be considered for *O*. *cyanea* in the WIO. The very divergent haplogroups in *O*. *vulgaris* from Brazil and Madagascar might be evolving towards speciation and therefore should be considered as separate species in FAO statistics.

## Supporting information

S1 Table GenBank accession number of generated octopus COI sequences from this study and those of previous study. (DOCX)

S2 Table Pairwise ϕ_ST_ values between populations of *Octopus cyanea* in Western Indian Ocean. (DOCX)
