# Supplementary material for: Cryptic diversity and limited connectivity in octopuses: Recommendations for fisheries management
Source: PLoS One. 2019 May 13;14(5):e0214748. doi: 10.1371/journal.pone.0214748 (PMC6513052; doi:10.1371/journal.pone.0214748)
Supplement: S1 Table — (DOCX) [file pone.0214748.s001.docx]

**S1 Table**: GenBank accession number of generated octopus COI sequences from this study and those of previous study.

| **Species** | **Number of Samples** | **Accession number** | | **Sampling location** | **Site name in this study** | **Reference** |
| --- | --- | --- | --- | --- | --- | --- |
| ***Octopus cyanea*** | 171 | MK593176 - MK593229, MK593244 - MK593340, MK593365 - MK593375, MK593396 - MK593404 | | Madagascar  Ramena, Nosy be, Morondava, Andavadoaka, Salary, Beheloke, Besambay, Maromena, Fort Dauphin, Tamatave, Sainte Marie | Ramena, Nosy be, Morondava, Andavadoaka, Salary, Beheloke, Besambay, Maromena, Fort Dauphin, Tamatave, Sainte Marie | This study |
|  | 20 | MK593376 - MK593395 | | Tanzania: Stone Town | Stone Town | This study |
|  | 38 | MK593230 - MK593243 | | Kenya: Kanamai | Shimoni, Kanamai | This study |
|  |  | MK593341 - MK593364 | | Shimoni, |  |  |
|  | 1 | GQ900741* | | Hawaii |  | [56] |
|  | 2 | AB430534*, AB430535* | | Japan: Ogasawara Island,  Okinawa: Nakagusuku |  | [27] |
| ***Octopus vulgaris*** | 30 | MK593421 - MK593450 | | Madagascar: Lavanono,  Fort Dauphin | Lavanono, Fort Dauphin | This study |
|  | 4 | DQ683208 - DQ683211 | | South Africa: Struisbaai,  Houtbaai | Struisbaai | [60] |
|  | 2 | DQ683212 - DQ683213 | | South Africa: Port Elizabeth | Port Elizabeth | [60] |
|  | 7 | DQ683214 - DQ683220 | | South Africa: Durban, Umhlanga | Durban | [60] |
|  | 1 | HM104262 | | South Africa: Port Elizabeth | Port Elizabeth | [89] |
|  | 1 | KJ605279 | | South Africa: Port Elizabeth | Port Elizabeth | [90] |
|  | 17 | KT008562* - KT008578* | | Italy: Sardinia |  | [13] |
|  | 1 | KF844026* | | Brazil: Cabo Norte |  | [91] |
|  | 4 | KF844027 - KF844030 | | Brazil: Bragança | North Brazil | [91] |
|  | 1 | KF844031* | | Brazil: Salvador |  | [91] |
|  | 3 | KF844032 - KF844034 | | Brazil: Rio de Janeiro | South Brazil | [91] |
|  | 1 | KF844035 | | Brazil: Jureia | South Brazil | [91] |
|  | 2 | KF844036 - KF844037 | | Brazil: Guaruja | South Brazil | [91] |
|  | 1 | KF844038 | | Brazil: Guaratuba | South Brazil | [91] |
|  | 1 | KF844039 | | Brazil: Paranagua | South Brazil | [91] |
|  | 2 | KF844040 - KF844041 | | Brazil: Cabo de Santa Marta | South Brazil | [91] |
|  | 11 | HQ908426* - HQ908436* | | Turkey |  | [92] |
|  | 5 | KC311408* - KC311412* | | Turkey |  | [93] |
|  | 13 | JX500627* - JX500639* | | Greece: Ikaria and Samos |  | ^$^1 |
|  | 3 | FN424379 - FN424381 | | Saint Paul & Amsterdam Island | Amsterdam Island | [6] |
|  | 3 | DQ683224 - DQ683226 | | Senegal | Senegal | [60] |
|  | 3 | DQ683221 - DQ683223 | | Spain: Galicia | Galicia | [60] |
|  | 23 | JX500655* - JX50065577* | | Spain: Galicia |  | ^$^1 |
|  | 3 | DQ683205 - DQ683207 | | Tristan da Cunha | Tristan da Cunha | [60] |
|  | 3 | AB430546* - AB430548* | | Japan: Hyougo, Akashi, Futami  Kanagawa, Misaki  East China Sea |  | [27] |
| ***Octopus oliveri*** | 1 | MK593420 | | Madagascar: Lavanono |  | This study |
|  | 1 | GQ900744* | | Hawaii, Oahu |  | [56] |
|  | 2 | KC848885*, KC848886* | | Hawaii, Oahu |  | ^$^2 |
|  |  | |  |  |  |  |
| ***Callistoctopus luteus*** | 10 | MK593405 - MK593414 | | Madagascar: Fort Dauphin |  | This study |
|  | 1 | AB385874* | | Vietnam: Nha Trang |  | [27] |
|  | 1 | AB430526* | | Japan: Okinawa, Ohdo Beach |  | [27] |
|  | 1 | KF489434* | | India: Kerala, Sakthikulangara |  | ^$^3 |
|  |  |  | |  |  |  |
| ***Callistoctopus ornatus*** | 5 | MK593415 - MK593419 | | Madagascar: Fort Dauphin |  | This study |
|  | 1 | AB430528* | | Japan: Okinawa, Ohdo Beach |  | [27] |
|  | 1 | AY616892* | | Australia |  | [94] |

^$1^ Jassoud, A.F.J., Lado Insua,T., Lucas,M., Miliou,A. and Schizas,N. Department of Marine Sciences, University of Puerto Rico, Mayaguez, USA, unpublished data

^$2^ Ylitalo,H. A. *et al*., Hawaii Institute of Marine Biology, 46-007 Lilipuna Rd, Kaneohe, HI 96744-3617, USA, unpublished data

^$3^ Appukuttan Nair, B. *et al*., University of Kerala, Puzhayoram, Amritha Nagar, Kaimanam, Thiruvananthapuram, Kerala 695581, India, unpublished data

*sequences used for NJ tree only

1. Strugnell JM, Norman MD, Vecchione M, Guzik M, Allcock AL. The ink sac clouds octopod evolutionary history. Hydrobiologia. 2014; 725: 215 – 235.
2. Amor MD, Norman MD, Cameron HE, Strugnell JM. Allopatric Speciation within a Cryptic Species Complex of Australasian Octopuses. PLoS ONE. 2014; 9: 1 - 13.
3. Sales JBDL, Rego PSD, Hilsdorf AWS, Moreira AA, Haimovici M, Tomás AR, et al. Phylogeographical features of *Octopus vulgaris* and *Octopus insularis* in the Southeastern Atlantic Based on the Analysis of Mitochondrial Markers. J. shellfish res. 2013; 32: 325–339.
4. Keskin E, Atar HH. Genetic divergence of Octopus vulgaris species in the eastern Mediterranean. Biochem. Syst. Ecol. 2011; 39: 277–282.
5. Keskin E, Atar HH. DNA barcoding commercially important aquatic invertebrates of Turkey. Mitochondrial DNA. 2013; 24: 440–450.
6. Strugnell J, Norman M, Jackson J, Drummond AJ, Cooper A. Molecular phylogeny of coleoid cephalopods (Mollusca: Cephalopoda) using a multigene approach; the effect of data partitioning on resolving phylogenies in a Bayesian framework. Mol Phylogenet Evol. 2005; 37: 426–441.
