# Supplementary material for: Cryptic diversity and limited connectivity in octopuses: Recommendations for fisheries management
Source: PLoS One. 2019 May 13;14(5):e0214748. doi: 10.1371/journal.pone.0214748 (PMC6513052; doi:10.1371/journal.pone.0214748)
Supplement: S2 Table — (DOCX) [file pone.0214748.s002.docx]

**S2 Table**: Pairwise φ_ST_ values between populations of *Octopus cyanea* in Western Indian Ocean**.** Underscored values are significant after sequential Bonferroni correction.

| **sites** | **Ka** | **Sh** | **St** | **Ra** | **No** | **Mo** | **An** | **Sa** | **Be** | **Bb** | **Ma** | **Fd** | **Ta** | **Sm** |
| --- | --- | --- | --- | --- | --- | --- | --- | --- | --- | --- | --- | --- | --- | --- |
| **Ka** | 0.00 |  |  |  |  |  |  |  |  |  |  |  |  |  |
| **Sh** | 0.08* | 0.00 |  |  |  |  |  |  |  |  |  |  |  |  |
| **St** | 0.02 | 0.00 | 0.00 |  |  |  |  |  |  |  |  |  |  |  |
| **Ra** | 0.03 | -0.00 | -0.01 | 0.00 |  |  |  |  |  |  |  |  |  |  |
| **No** | 0.01 | -0.06 | -0.06 | -0.06 | 0.00 |  |  |  |  |  |  |  |  |  |
| **Mo** | 0.07* | -0.02 | -0.01 | -0.02 | 0.00 | 0.00 |  |  |  |  |  |  |  |  |
| **An** | 0.01 | 0.01 | -0.01 | -0.01 | -0.06 | -0.01 | 0.00 |  |  |  |  |  |  |  |
| **Sa** | 0.02 | 0.01 | 0.01 | 0.00 | -0.04 | -0.00 | -0.01 | 0.00 |  |  |  |  |  |  |
| **Be** | 0.01 | 0.19** | 0.11* | 0.14** | 0.10 | 0.20** | 0.05 | 0.08 | 0.00 |  |  |  |  |  |
| **Bb** | 0.06** | 0.02 | 0.01 | 0.01 | -0.04 | 0.00 | 0.01 | 0.02 | 0.14* | 0.00 |  |  |  |  |
| **Ma** | -0.00 | 0.05* | 0.02 | 0.04 | -0.01 | 0.04 | -0.01 | -0.01 | -0.01 | 0.05* | 0.00 |  |  |  |
| **Fd** | 0.12 | 0.50 | 0.11 | 0.28 | 0.63 | 0.79* | 0.05 | 0.33 | 0.10 | 0.30 | 0.17 | 0.00 |  |  |
| **Ta** | 0.03 | 0.04 | -0.03 | 0.01 | -0.01 | 0.07 | -0.03 | 0.02 | 0.06 | 0.02 | -0.02 | 0.02 | 0.00 |  |
| **Sm** | 0.03 | 0.04 | -0.01 | 0.02 | -0.03 | 0.04* | -0.01 | 0.03 | 0.06 | 0.03 | 0.01 | -0.04 | -0.10 | 0.00 |

For abbreviations of samples sites see Table 1. Signiﬁcance levels: *0.05 > P > 0.01; **P ≤ 0.01; all other values not signiﬁcant (P ≥ 0.05).
